# Importance of Community Health Workers for Maternal Health Care Management

**DOI:** 10.3389/phrs.2024.1606803

**Published:** 2024-02-22

**Authors:** Archana Gupta, Saba Khan

**Affiliations:** Department of Home Science, Faculty of Agricultural Sciences, Aligarh Muslim University, Aligarh, India

**Keywords:** Anganwadi workers, ASHA workers, community health workers, lactating mothers, pregnant women

## Abstract

**Objectives:** Community Health Workers (CHWs) are important healthcare professionals and key members of team. The purpose of this research is to identify the roles and responsibilities of CHWs in developed and developing countries who provide healthcare assistance to pregnant and lactating women.

**Methods:** For this particular study, a comparison was conducted between CHWs role in seven developed countries, seven South Asian developing countries, and India, with special emphasis on improving maternal health status.

**Results:** CHW programs are essential in communities, institutional health programs, and outreach delivery systems. Without active community involvement, CHWs cannot reach their full potential. Developed countries have frameworks for CHWs, such as the Swasthya Shebika Program, Village Health Worker Cadret, Lady Health Worker Programme, and Accredited Social Health Activist program. CHWs are well-paid in developed nations and work with marginalized groups to spread health messages. However, up to 60% of community health workers in low- and lower-middle-income countries do not receive remuneration.

**Conclusion:** Health systems must support CHWs in choosing technical interventions and providing necessary training, supervision, and logistical support.

## Introduction

“Communities” are groups of people who may or may not live close to one another but who still hold the same beliefs, concerns, or identities as per WHO. These groups may be local, national, or international and may have narrow or broad interests. The term “community health worker” (CHW) refers to social service and/or public health professionals who work effectively with and for the community, promoting healthy lifestyles. They might provide paid or unpaid volunteer work for a nearby business, institution, or hospital. Similarities in race, language, culture, financial background, values, and life experiences exist between CHWs and the community individuals they serve. According to the US Department of Labour, CHWs are those who “help individuals and communities adopt healthy behaviors” and “conduct outreach” in addition to “advocating for people’s individual and community health needs” [1].

Health of women during pregnancy, childbirth, and the postpartum period is referred to as maternal health. Every stage should be healthy in order to help women and children achieve their greatest potential for wellbeing. A shocking 287,000 women died during and after pregnancy and delivery in 2020, despite tremendous progress over the previous two decades [2].

The Millennium Development Goals (MDGs), which were completed in 2015, were replaced with the Sustainable Development Goals (SDGs), which are intended to be accomplished by 2030 [3]. The SDG states that by 2030, the neonatal mortality rate should be as low as 12 per 1,000 live births and the MMR (maternal morality ratio) should be reduced by 70 per 100,000 live births. The provision of primary healthcare is hampered by insufficient government spending, a shortage and unequal distribution of the health workforce, and inadequate multi sectoral training [4]. At this time, WHO started to place a fresh emphasis on primary healthcare (PHC) and driven Universal Health Coverage (UHC) as a means of achieving the SDGs related to health.

The National Health Mission (NHM) [5] has two sub-missions: the National Rural Health Mission (NRHM) and the National Urban Health Mission (NUHM). The three main elements of the programme are Communicable and Non-Communicable Diseases, Reproductive, Maternal, Neonatal, Child, and Adolescent Health (RMNCH+A), and. Everyone will have access to fair, affordable, and high-quality healthcare services that are responsible and considerate of the needs of their patients, in accordance to the NHM. UNICEF works with the Ministry of Health and Family Welfare (MoHFW), the Ministry of Women and Child Development (MWCD), NITI Aayog, and state governments to support planning, budgeting, policy creation, capacity building, monitoring, and demand generation. It supports the abilities of health administrators and supervisors at the district and block levels when planning, implementing, monitoring, and supervising efficient maternal healthcare services with a focus on high-risk pregnant women and those in hard-to-reach, vulnerable, and socially disadvantaged communities. Reaching every mother, providing continuum of care, and providing antenatal care are among the measures by the Indian government that UNICEF is supporting.

UNICEF supports the implementation of the MoHFW’s policy, which stipulates that every delivery should be attended by a qualified healthcare professional at a hospital in order to enhance the health and nutrition of pregnant mothers and provide high-quality maternal health services. To guarantee a healthy pregnancy and rapidly identify high-risk conditions that could harm their health or the health of their growing foetus, all expectant mothers must register for antenatal treatment at the local healthcare institution promptly as they become aware of their pregnancy.

To achieve the global goal of improving maternal health and saving women’s lives, we need to accomplish more to reach those who are most at risk, such as women in rural areas, urban slums, households with fewer resources, adolescent mothers, women from minorities and tribal, Scheduled Caste, and Scheduled Tribe groups.

The significance of study is multiple folded. Firstly, it will enable in exploring the problems faced by health workers and beneficiaries in implementing maternal healthcare schemes or programme generated by government. This also enables in knowing nations perspectives regarding maternal healthcare. Understanding more about the precise role, responsibilities, and contributions of these CHWs in enhancing the health status of the community. This will also be helpful to the larger community, government, policymakers, health planners, national programme, researchers, programme managers, and the public health system of India. Recognizing the promoters and obstacles that arise in meeting the community’s fundamental health needs can also be helpful in future efforts to advance their work and social standing and consequently build a healthy nation.

## Methods

The present study “Importance of Community Health Workers for Maternal Health Care Management” is undertaken with the objective to understand nations perspectives regarding maternal healthcare. Also, to review the roles and responsibilities of community health workers and recognize the obstacles arising while reaching community health needs.

**FIGURE 1:**
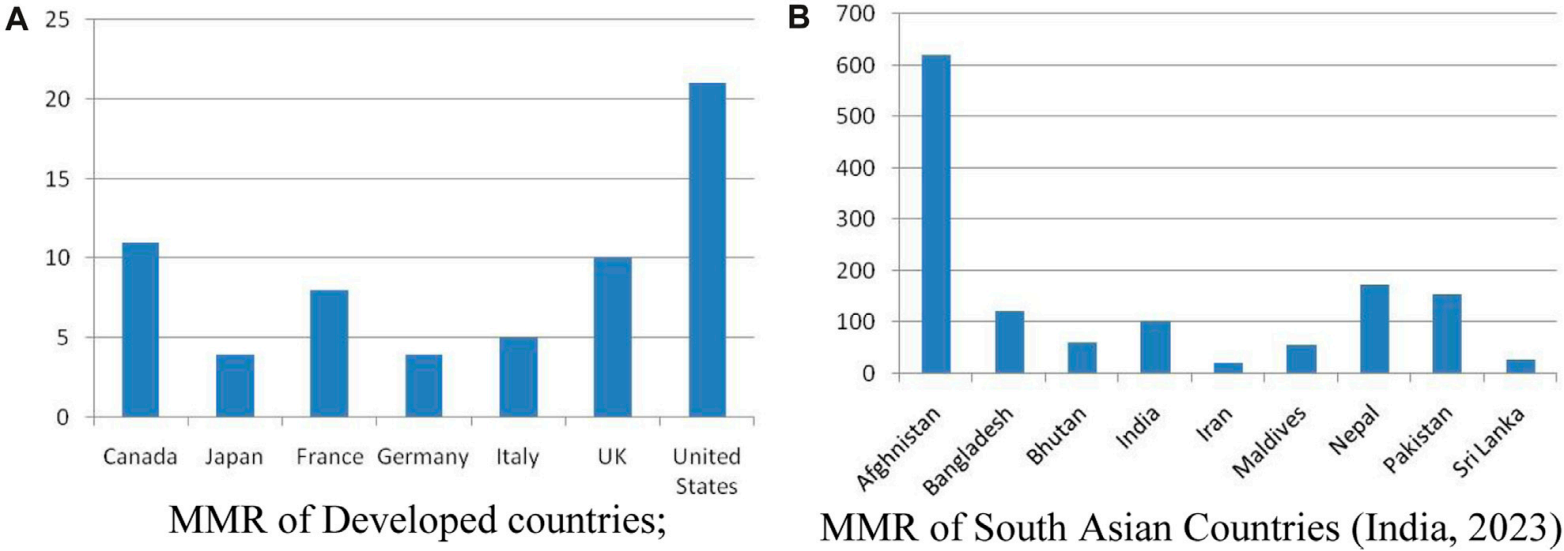
**(A)**: MMR of Developed countries; **(B)** MMR of South Asian Countries (India, 2023).

Maternal mortality rate is much higher in developing countries than in developed countries as presented in Figures 1A, B. For this reason, the methodology accepted for the particular study includes CHWs and their roles in seven developed countries and seven south Asian developing countries were compared in addition with Indian studies on contributing roles of community health workers in improving maternal health status.

All nations are divided into three groups based on their economics, according to the World Economic Situation and Prospects (WESP) classifications (2022): established economies, economies in transition, and emerging economies. Seven significant developed nations and South Asian developing nations were chosen for the current study.

Iran being a South Asian developing country was not purposely selected because its MMR is 22 per 100,000 live births. With a maternal mortality rate (MMR) reduction of 75% by 2015, Iran is one of the nations that has succeeded in fulfilling MDG5, having attained the largest reduction among its neighbors, with the exception of Turkey. Iran’s MMR was 274 in 1975, dropped to 150 in 1990, continued to decline, and reached 94 in 1995, 38 in 2005, 30 in 2008, and 25 in 2015, a number comparable to developed countries [6, 7].

A review of the many advancements made by the community health workers (CHWs) in basic healthcare is presented in this paper. This can be used as a valuable resource for learning about the work carried out by CHWs to ensure high-quality healthcare delivered to the target population by reviewing it. This literature review was conducted with the assistance of databases like PubMed, Google, and Google Scholar in order to gather a comprehensive amount of information. As part of this study, we investigated how CHWs are able to efficiently deliver vital healthcare services to the community.

## Results

In this section, Figures 1A, B show the maternal mortality ratio (modelled estimate, per 100,000 live births) as reported by WHO [2], UNICEF, UNFPA, the World Bank Group [8], and UNDESA/Population Division.

From the data presented in Figure 1 this is revealed that Iran being an developing country has MMR equivalent to developed countries. The 1920s saw the establishment of a sizable CHW programme in Ding Xian, China. At that time, Jimmy Yen, a Chinese community development specialist with experience teaching adult literacy, and Dr. John B. Grant, a Rockefeller Foundation employee assigned to Peking Medical University, trained illiterate farmers to keep birth and death records, administer smallpox and other disease vaccinations, give first aid and health education talks, and assist communities in keeping their wells clean [9].

The CHW projects in South Asia have a long history of being active, and the CHWs there still play a significant part in primary healthcare and serve as links between the community and the healthcare system. Countries in the region are simultaneously experiencing demographic and epidemiological changes, with populations that are getting older, more urbanized, and suffering from a greater burden of non-communicable diseases. In such a situation, it is vital to increase the contribution of CHW initiatives to PHC strengthening and fulfilling the region’s objectives and aspirations.

### Roles and Responsibilities of CHW

In this section, roles and responsibilities of Community health workers is discussed with special emphasis on their implementation in the community, training, salary and impact on the society. Seven major developed countries - Canada, Japan, France, Germany, Italy, United Kingdom and United States were selected to review the roles and responsibilities of CHWs in developed countries. The roles, responsibilities and implementation of CHWs in respective countries is presented in Table 1.

**TABLE 1:** Roles and responsibilities of CHWs in major developed countries (India, 2023).

S.No	Country	Background	Implementation	Training	Roles and responsibilities	Incentives	Supervision	Impact
1	Canada [11]	The CHW Network of Canada offers a space where CHWs and their allies can exchange information, skills, and resources. CHWs are rooted in the communities they serve and go by various names	CHWs are employed in a variety of settings, such as public health departments, community health facilities, organisations with a focus on certain ethnic groups and cultures	Organisational training programmes, institutional training programmes, and on-the-job training are the three types of education and training	HIV prevention, maternal-child and nutritional health promotion, oral health promotion for preschoolers, and facilitating marginalised communities’ access to health services	In Canada, a community health worker makes $51,097 years, or $26.20 per hour	CHWs work under different divisions—Hamilton’s Ontario Public Health division, Multicultural Health Team, Ottawa Public Health and the Toronto Public Health Unit	CHWs reach out to marginalised populations with targeted programmes and important health messages and are well-paid with good benefits
2.	Japan [12, 13]	Health promotion volunteers in Japan focus on supporting the elderly and educating future generations about disease prevention	CHW intervention in Japan has changed over time to focus on preventing chronic diseases associated with aging and staving off frailty among the elderly	CHWs are trained by municipality generally for lasts between 1 and 6 months of duration for providing awareness among community people regarding healthy diet, maternal and child health and proper development	Major role of HW is shifted towards providing education, awareness and counselling regarding various health issues and non-communicable diseases to community members	Generally are unpaid volunteers	Supervised by municipality	CHWs contributes to increasing the number of healthy years lived and decreasing health inequalities
3.	France [14, 15]	Midwives are the health professionals who work to improve maternal health status	Midwives along with other professionals are implemented to focus on pregnant and lactating women	France offers comprehensive training for health careers, covering 5 years, including midwifery education in 35 schools across Europe	Midwives maintain surveillance on women during pregnancy and childbirth, and they help new mothers by offering postnatal care	They work in private or public sector; and some are also self employed	Supervised by European midwifery associations	Improves quality care for all women and newborn infants
4.	Germany [16]	The public and private sectors both use midwives’ services	Despite the fact that childbirths in Germany take place in hospitals, midwives are required to be present at every birth and can also provide services in women’s homes	Either a 4-year degree from universities of applied sciences or a 3-year basic vocational education	Midwives are assigned role of providing assistance during pregnancy, birth and postpartum duration	Depending on number of hours, they are paid by management of organization	Supervised by federal and state laws which support and secure their practice	Majority of pegnant women received essential integrated cares and schemes awareness
5.	Italy	Healthcare workers and midwives provide support to pregnant women during motherhood, delivery and childcare	Health professionals are organized under Ministry of Health and run as a universal public healthcare system	Training lasts 3 years including internship periods in healthcare facilities	Health professionals are crucial in providing family planning advice and reducing illness transmission from mother to child. Health professionals are crucial in providing family planning advice and reducing illness transmission from mother to child	Healthcare workers earn minimum of 1,191 to 3,383 EUR per month	Supervised by Ministry of Health	Significant improvement in the health of pregnant women and child health
6.	United Kingdom [17]	Healthcare work professionals provide parenting classes, clinical examinations and screening	Health professionals are implemented by NHS (National Health Service), England	Broad structured curriculum with a low level of technical proficiency	Health promotion for behavioral changes, including quitting smoking, encouraging breastfeeding, maintaining good sexual health, and engaging in physical activity	In the United Kingdom, a healthcare professional makes an average annual pay of £22,078	Many courses and training programs are available to enable them to provide good maternal health support	Health professionals improves the perinatal and postpartum outcomes
7.	United States [18, 19]	CHWs reach formerly to under served individuals that often have complicated medical and social requirements	CHWs were implemented in US with aim to reduce life threatening complications in pregnant women	Currently, 30 authorities provide CHWs with some kind of standardized training	Home visits, health-promoting initiatives, and the prevention of ailments like TB and HIV	CHWs receive a set monthly salary of SLL 150,000 ($17.5), while supervisors are compensated with SLL 250,000 ($29.2)	CHW supervisor plays a crucial role in providing counselling and support to CHWs	Risks of diabetes and hypertension was reduced on intervention of CHWs

This can be summed from Table 1, that CHW in developed countries are provided with a framework to discuss their knowledge, challenges, resources and implementations to be applied to create a sustainable growth in terms of maternal health, child care and elderly support. Midwives along with other professionals are implemented to focus on pregnant and lactating women.

CHW are implemented by NHS (National Health Services) with the aim to reduce complications associated with pregnant and lactating women. Health professionals are organized under Ministry of health and run as universal public healthcare system.

In addition to professional education additional training of varied duration 3 months, 6 months or 1 year is provided to increase on job training experience. Various awareness programme are launched to increase knowledge regarding challenges faced by vulnerable group of people (pregnant mothers, lactating women, infants and elderly people). France offers comprehensive training for health careers, covering 5 years, including midwifery education in 35 schools across Europe.

Major role of CHW is shifted towards providing education, awareness and counselling regarding various health issues and non-communicable diseases to community members. HIV prevention, maternal-child and nutritional health promotion, oral health promotion for preschoolers, and facilitating marginalized communities’ access to health services. Despite the fact that childbirths in Germany take place in hospitals, midwives are required to be present at every birth and can also provide services in women’s homes.

CHWs reach out to marginalized populations with targeted programme and important health messages and are well-paid with good benefits. The expense of this care is covered by both statutory and private health insurance providers [10]. CHW are paid by the management of organization depending on the sector whether public or private sector. Additional incentives and benefits are also provided.

Majority of CHWs are supervised by Ministry of Health. Various awareness and training programmes are implemented under the supervision of organizations to enhance the impact of CHW on beneficiaries. CHWs improve health outcomes by reaching marginalized communities, reducing health inequalities, and promoting quality care for women and newborns. They also reduce diabetes and hypertension risks through integrated care and awareness programs.

The background information, roles and responsibilities of CHWs in seven Sub Asian developing countries—Afghanistan, Bangladesh, Bhutan, Maldives, Nepal, Pakistan, Sri Lanka is presented in Table 2.

**TABLE 2:** Roles and responsibilities of CHWs in Sub Asian Developing countries (India, 2023).

S.No	Country	Background	Implementation	Training	Roles and responsibilities	Incentives	Supervision	Impact
1.	Afghanistan	The community-based healthcare (CBHC) a portion of the Basic Package of Health Services (BPHS) includes the Community Health Workers (CHW) project	In order to execute BPHS, CHWs are stationed at health posts as male and female teams, working in pairs	CHWs receive a month of field experience in their villages between three separate 3-week modules	CHWs provide a comprehensive set of services from health promotion to referral to the next level of care, including community case management, TB treatment, and family planning commodities	CHW are volunteers	Community Health Supervisors (CHSs) visit health posts and CHWs meet monthly to discuss health issues	CHWs are an important contributor to Afghanistan’s improved health status in the past decade.
2.	Bangladesh [20–24]	In order to address socio-cultural barriers, Bangladesh has a history of deploying CHWs to support health services, with BRAC’s Swasthya Shebika Programme concentrating on female health workers	The number of SSs has grown significantly since 1990 from 70,000 to more than 100,000	BRAC provides basic training to SSs for 4 weeks on how to manage illnesses, encourage healthy habits, and refer patients to services	In addition to educating families on nutrition, safe childbirth, FP, immunisations, cleanliness, and water and sanitation, SSs offer health promotion programmes. In order to give Community Health Volunteers CHVs an additional incentive in addition to the sales component, BRAC created it	To create revolving money and market health items with a minimal markup, CHVs use modest loans	Swasthya Kormis (SKs) provide direct supervision to CHW	The programme in Bangladesh has significantly lowered under-5 mortality and improved TB control on a countrywide level
3.	Bhutan [25]	The CHW cadre in Bhutan is the Village Health Worker (VHW), who provide primary healthcare in the country. VHWs are responsible for providing first aid and treatment for minor ailments, creating awareness, and mobilising the community for health promotion	The MoH is responsible for policy formulation and implementation, while the (District Health Management Team) DHMT is responsible for providing health services and responding to local needs. The (Non-Communicable Disease Division) NCDD is responsible for maintaining the VHW database. Health administration and management are devolved to districts and MOH provides technical support to local governments	VHA receive training on interpersonal communication by Health Assistant (HA) and DHO. 3 days refresher trainings are given annually	VHWs promote healthy practices and care seeking behaviours in the community, facilitating access to quality services	VHWs are voluntary cadres with no salary and limited monetary incentives for training and immunization campaigns	HAs in health facilities are responsible for training and supervising VHWs. Community members and the Gewog Yargay Gewog Tshogde (GYT) also monitor the VHWs’ activities, but supervision is generally poor with no standard operating procedures or clear guidance	Gender imbalance in VHWs could affect the uptake of health services by women and adolescent females. To increase female participation, cultural, social and gender barriers must be addressed, as well as improving employment conditions
4.	Maldives [26]	The two different cadres of CHWs in the country are the Family Health Worker (FHW, or Family Health Officer), and the Community Health Worker (CHW, or Community Health Officer). Assistant, CHO, and Senior CHO are the three different ranks of CHOs	CHWs provide a range of public health, preventative, and curative services, particularly those pertaining to reproductive health, out of the atoll hospital’s Public Health Unit or an island-level health clinic. Formerly based in the community, Family Health Workers (FHWs) are now connected to the Health Centre but continue to conduct outreach and home visits	18 months advanced course in Primary Healthcare (PHC), 30 months course in diploma and 4years Bachelor’s degree course	With the aid of CHWs, up to fifteen national public health programmes are put into action. A nutritional programme that incorporates growth monitoring, vaccination, NCD assessment, health education, communicable disease prevention and promotion activities, reproductive health and family planning, prevention and monitoring of diarrhea, and surveillance and control of vectors	The MoH employs CHW and FHW cadres as official salaried government employees who are completely integrated into the civil service system	The provision of frequent oversight and monitoring was hampered by a number of problems, including inadequate finance, a lack of technical and supervisory employees and staff turnover, as well as geographical obstacles. This has an effect on the provision of high-quality services and helps explain the inadequate adherence to and accountability for national standards	The strategic orientations for coordinating primary and preventive healthcare, public health services, and the creation of a variety of care services, from promotion and prevention to treatment and rehabilitation, were discussed under the guidance of the MPHF
5.	Nepal [27–29]	VHWs, MCHWs, and FCHVs (Female CHVs) collaborate to assist each other’s areas of responsibility and are based in neighbourhood health centres. While FCHVs provide logistical support for the distribution of vitamin A, VHWs organise communities for immunisation	Each health centre employs one professional health worker in addition to one VHW, one MCH Worker (MCHW), and normally nine (but possibly more) Female CHVs (FCHVs) to serve a region with a population of 5,000–10,000 people	FCHVs receive initial training for 18 days, followed by 5-day refresher training every 5 years	CHWs have defined scope of work, with MCHWs offering reproductive services, VHWs offering family-oriented services, and FCHVs providing basic services and health education	The government formally employs and compensates MCHWs and VHWs for their work. Non-financial incentives like a clothes allowance and community recognition are motivating elements for FCHVs	The FCHVs who operate in their geographic regions are supervised by VHWs and MCHWs. During monthly supervision visits, VHWs and MCHWs are in charge of replenishing the FCHVs and giving support, counsel, and feedback	In regard to low-income nations, Nepal has led the world in lowering its MMR, under-5 death rate, and fertility rate. There is broad consensus that Nepal’s CHWs, especially the FCHVs, have contributed significantly to the accomplishment of the significant objectives—child and maternal health
6.	Pakistan [25, 27, 30]	In order to provide primary care services to underserved populations, the Lady Health Worker Programme (LHWP) was started in 1994. Improving service quality and broadening LHWP coverage were the two objectives outlined in the national strategic plan of 2003	In Pakistan, LHWs run so-called “Health Houses,” which serve as emergency care and treatment facilities. They are in charge of about 1,000 people, with young children and couples in active reproduction receiving priority	LHWs undergo a year of on-the-job training, along with a week of refresher training every year, but training schedules vary by province	LHWs visit 27 houses each week and offer services like MCH, newborn care, TB community management, and HIV/AIDS health education	LHWs receive a salary of about $343 per year. Because it frequently acts as the only source of income, the LHW stipend is an essential source of support for families	An LHW Supervisor (LHS) oversees each LHW each month. They are all connected to public health clinics.	For Pakistan’s LHW Programme to provide adequate service coverage on a national scale for MCH SDGs, it must be improved and expanded
							LHSs should visit with clients and LHWs for community-based supervision at least once a month. The LHSs should use this time to assess the LHWs’ work and plan their own workday for the upcoming month.	
7.	Sri Lanka [31]	In Sri Lanka community health workers have two key frameworks that are currently active—Public Health Midwife (PHM) and Public Health Inspector (PHI)	The midwife curriculum in Sri Lanka is based on WHO and ICM competency frameworks, with content covering family planning, nutrition education, antenatal care, care of children with special needs, and psychosocial and reproductive needs of young widows	The 18-month training period has remained unchanged since the abolition of PHM was founded 93 years ago. A maximum of 3 years or a degree program was suggested to be adopted for PHM recommendation to provide “better care”	They have prevntive, promotive and curative role. Promotes antenatal care, immunization, vaccination, breast feeding, maternal health, etc.	PHM and PHI compensation is aligned with government salary structures and in general salaries are paid on time. They also get accommodation, maintenance and allowances such as transport and communication subsidies	The monitoring and evaluation unit of MoH FHB monitors the implementation of the RMNCH to the program, including the operation of PHM	Sri Lanka achieved a high Human Development Index in 2019 and its health system has been recognized as successful, contributing to the attainment of the Millennium Development Goals

CHWs are essential components of the Basic Package of Health Services (BPHS) community-based healthcare (CBHC) section. Bangladesh has a history of using CHWs to support health services, with BRAC’s Swasthya Shebika (SS) Program focusing on female health workers. Bhutan has a Village Health Worker (VHW) cadre, while Family Health Worker (FHW) and Community Health Worker (CHW) cadres work together. The Lady Health Worker Programme (LHWP) was established in 1994 to provide primary care services to underserved populations. In Sri Lanka, community health workers have two key frameworks: Public Health Midwife (PHM) and Public Health Inspector (PHI).

Health centres, including health centres, employ a District Health Management Team (DHMT) and NGOs to implement BPHS. The MoH is responsible for policy formulation and implementation, while NGOs provide health services and support local needs. Family Health Workers (FHWs) are associated with health centres, providing outreach and house visits. In Pakistan, LHWs provide emergency care at their homes, with priority given to reproductive couples and children under five. Sri Lanka’s midwife curriculum is based on WHO and ICM competency frameworks, covering family planning, nutrition education, antenatal care, and psychosocial and reproductive needs of young widows.

CHWs receive a month of field experience, while SSs receive 4 weeks of basic training, VHA receives interpersonal communication training, and receive 3 days of refresher training annually. FCHVs receive 18 days of initial training, followed by 5-day refresher training every 5 years. LHWs receive 1 year of on-the-job training, including 1 week of refresher training each year. The 18-month training period remains unchanged since PHM abolition 93 years ago.

CHWs offer health promotion, referral, and referral services, including community case management, TB treatment, and family planning. They educate families on nutrition, safe delivery, immunizations, hygiene, and water and sanitation. CHWs implement up to fifteen national public health programs, including nutrition, NCD screening, communicable disease prevention, reproductive health, family planning, and vector surveillance. They provide reproductive services, family-oriented services, and basic health education. LHWs visit 27 households weekly, promoting antenatal care, immunization, vaccination, breastfeeding, and maternal health. A nutrition programme that includes growth monitoring and immunisation, NCD screening and health education, communicable disease prevention and promotion activities, reproductive health and family planning, prevention and surveillance for diarrhoea, and vector surveillance and control is regulated or coordinated by community health workers.

CHW and VHW are voluntary cadres with limited monetary incentives for training and immunization campaigns. The MoH employs CHW and VHW as official government employees, with government compensation and non-financial incentives. LHWs earn $343 a year, with a stipend being crucial for family assistance. Compensation aligns with government salary structures, and they receive accommodation, maintenance, and allowances.

The MoH is responsible for policy formulation and implementation, while the (District Health Management Team) DHMT is responsible for providing health services and responding to local needs. Community Health Supervisors (CHSs) and Swasthya Kormis provide monthly supervision to health posts, while HAs train and supervise VHWs. However, poor supervision and monitoring due to insufficient funding, staff turnover, and geographical barriers hinder quality service provision and compliance with national guidelines. FCHVs are supervised by VHWs and MCHWs, while LHWs are affiliated with public health clinics and oversee them monthly. LHSs review work and create work schedules, while the MoH FHB monitors the implementation of RMNCH and PHM operations. Insufficient funding, shortages and turnover of technical and supervisory staff, and geographical barriers were some of the factors hampering the provision of regular supervision and monitoring [32].

CHWs have significantly contributed to Afghanistan’s improved health status, reducing under-5 mortality and national TB control. Addressing gender imbalance in VHWs and improving employment conditions is crucial for female participation. Nepal has led the world in lowering maternal mortality, under-5 death rate, and fertility rates, while Pakistan needs to expand and strengthen its LHW Program for proper service coverage. Sri Lanka’s health system has achieved high Human Development Index in 2019 and contributed to the attainment of the Millennium Development Goals.

The roles and responsibilities of CHWs in India is presented in Table 3 with special emphasis on Auxiliary Nurse and Midwife (ANM), Accredited Social Health Activist (ASHA), Anganwadi workers (AWW).

**TABLE 3:** Roles and responsibilities of CHWs in India (India, 2023).

S.No	Country	Background	Implementation	Training	Roles and responsibilities	Incentives	Supervision	Impact
1.	India [33–36]	India has three CHW cadres: ANM, AWW and ASHA. ANM provides care at subcenters, AWW works in villages and provides food supplements, and ASHA promotes MCH, including immunizations and deliveries	The ASHA program in India has been challenged due to the rigid hierarchical structure of the health system, despite its design to interact with the formal healthcare system and communities	AWWs and ASHA employees each receive 3–4 weeks of training, whereas ANMs receive 18 months with occasional additional trainings	ANMs are now MPWs with responsibilities to manage birth complications. AWWs manage nutritional supplementation and promote healthy behaviors, while ASHA workers focus on institutional deliveries, immunizations, provision of basic medicines, and referral of patients to the subcenter	ANMs are paid a government salary, while AWWs are volunteers paid an “honorarium.” ASHA workers also receive performance-based incentives for facilitating institutional deliveries and immunizations	All the three cadres of CHWs are supervised individually	Evaluations of CHW programs have produced mixed results, but efforts are being made to improve training, supervision, remuneration, and logistical support

In India the National Rural Health Mission (NRHM) was developed in 2000 to improve rural PHC, accountability, and community engagement in the public health sector. In 2005, the Accredited Social Health Activist (ASHA) program was launched with motivating and recognition initiatives. The ASHA Facilitator is responsible for developing health reports and consolidating information about pregnancies, births, deliveries, newborn care, and deaths. By cultivating a sizable pool of CHWs known as Accredited Social Health Activists (ASHAs), the Indian National Rural Health Mission (NRHM) 2005 sought to enhance health outcomes.

Family planning, bringing expectant women to hospitals for birth, mother and child health, and health education are among the tasks. They travel with a first aid pack. ASHAs are assigned 200 families, with a total population of roughly 1,000 people, to work in both urban and rural regions.

A 23-day training programme meant to provide ASHA workers with the required knowledge and abilities. ASHAs are honorary volunteers given honorarium and performance-based incentives. States can design their own incentives, with some introducing fixed monthly honorariums. Starting 2018, ASHAs will receive a minimum of Rs. 2,000/- per month for routine activities, along with other task-based incentives approved at Central/State level.

All three workers are supervised individually. THe impact of ANM, ASHA and AWW is positive in nature. They promote better utilization of available schemes by the people. Also help in reducing MMR (Maternal Mortality Rate) and IMR (Infant Mortality Rate).

## Discussion

The evidence presented here indicates conclusively that CHW programs are not self-sustaining entities. Instead, they play a significant role in a larger system of activities that includes communities, institutional health programs, and particular interventions that call for an outreach delivery system. Without the active involvement of communities as partners in collaboration and support, CHWs cannot reach their full potential. In order to choose the proper technical interventions for CHW programs and to give them the training, supervision, and logistical support that these interventions need, health systems must fully support them.

According to the 2006 World Health Report, countries that have fewer than 2.28 physicians, nurses, and midwives per 1,000 residents typically do not meet targeted 80% coverage for child immunization and competent birth attendance. The proportion of health professionals in the population and the survival of pregnant women and young children are directly correlated. Survival decreases proportionally when the number of health professionals decreases. The improvement of the nation’s condition depends on the protection and promotion of women’s health, that demands for a multisectoral plan of action. In developed countries, health services are provided with free comprehensive coverage to entire population irrespective of socioeconomic status in order to ensure equity in system.

The data show a noticeable rise in the number of health-related human resources, including midwives in Iran [37]. Approximately 33,208 midwives work in the Iranian health system [37] at various levels of management (Ministry of Health and Medical Education at the policy making and management level), education (training midwifery undergraduates, masters, and doctorates, and health workers), and as a member of the healthcare team and under the supervision of obstetricians or general practitioners in health centers [38, 39]. Midwives are the largest group of healthcare providers in health centres in Iran [40].

CHWs in developed countries are provided with a framework to discuss knowledge, challenges, resources, and implementations for sustainable growth in maternal health, child care, and elderly support. They are essential components of the Basic Package of Health Services (BPHS) community-based healthcare section. Bangladesh, Bhutan, Sri Lanka, and India have established frameworks for CHWs, such as the Swasthya Shebika Program, Village Health Worker Cadret, Lady Health Worker Programme, and Accredited Social Health Activist (ASHA) program.

In developed countries CHW are implemented by National Health Service whereas in developed counties by Ministry of Health. Preventing unintended pregnancy is essential if maternal deaths are to be avoided. All women must have access to legal, safe abortion services as well as high-quality post-abortion care. As long as complications are managed or prevented, the majority of maternal deaths can be avoided. All women must have access to high-quality care during pregnancy, labor, and the postpartum period. The possibility that poor women in rural areas will receive adequate healthcare is the lowest [41]. According to the most recent data, 99% of newborns in high- and upper-middle-income nations benefit from the presence of a qualified midwife, doctor, or nurse. But just 68% of low-income countries and 78% of lower-middle-income countries receive such skilled assistance [42].

ASHA workers receive basic training, refresher training, and field experience in developing countries. They receive a 23-day program to provide knowledge and abilities. Community health workers, answerable to communities, represent the essential “missing link” between societal yearnings and communities in need. They receive interpersonal communication training, refresher training, and on-the-job training, with the 18-month training period unchanged since PHM abolition 93 years ago.

In developed nations, CHWs are well-paid with excellent benefits and work with marginalised groups to spread important health messages and specific programmes. Both public and commercial health insurance companies pay the cost of this care. Depending on the sector—public or private—CHW are paid by the administration of the organisation. There are also additional rewards and incentives offered. However, alternative estimates—those looking at all community health workers rather than just those performing services on behalf of the government—show that in developing countries, up to 60% of community health workers in low- and lower-middle-income countries did not receive any remuneration. In India, ASHA workers rely mainly on the incentive they receive by registering pregnant women for JSY (Janani Suraksha Yojana).

The following are some factors that discourage women from seeking or receiving care during pregnancy and childbirth:

• Social determinants, such as income, access to education, race and ethnicity, that put some sub-populations at greater risk;
• harmful gender norms and/or inequalities that lead to discrimination; and health system failures that lead to 1) poor quality of care, including disrespect, mistreatment, and abuse, 2) insufficient numbers of adequately trained health workers, 3) shortages of essential medical supplies, 4) the poor accountability of health systems.

Barriers to accessing high-quality maternal health treatments must be identified and removed at the health system and social levels in order to promote mother health in developing economy countries so as to achieve sustainable development goals.
